# The Experiences of Functioning and Health of Patients With Primary Sjögren's Syndrome: A Multicenter Qualitative European Study

**DOI:** 10.3389/fmed.2021.770422

**Published:** 2021-11-18

**Authors:** Julia Unger, Malin Mattsson, Răzvan G. Drăgoi, Claudiu Avram, Carina Boström, Frank Buttgereit, Angelika Lackner, Torsten Witte, Bernd Raffeiner, Peter Peichl, Martina Durechova, Josef Hermann, Tanja A. Stamm, Christian Dejaco

**Affiliations:** ^1^Department of Rheumatology and Immunology, Medical University of Graz, Graz, Austria; ^2^Department of Health Studies, Institute of Occupational Therapy, University of Applied Sciences FH JOANNEUM, Bad Gleichenberg, Austria; ^3^Division of Physiotherapy, Department of Neurobiology, Care Sciences and Society, Karolinska Institutet, and Affiliated to Women's Health and Allied Health Professionals Theme, Medical Unit Occupational Therapy and Physical Therapy, Karolinska University Hospital, Stockholm, Sweden; ^4^Department of Physiotherapy, Sunderby Hospital, Luleå, Sweden; ^5^Department of Balneology, Rehabilitation Medicine and Rheumatology, “Victor Babeş” University of Medicine and Pharmacy Timisoara, Timisoara, Romania; ^6^Department of Physical Therapy and Special Motility, West University of Timisoara, Timisoara, Romania; ^7^Department of Rheumatology and Clinical Immunology, Charitè University Medicine, Berlin, Germany; ^8^Department of Rheumatology and Immunology, Medical University of Hanover, Hanover, Germany; ^9^Department of Rheumatology, Hospital of Bolzano (SABES-ASDAA), Bolzano, Italy; ^10^Evangelic Hospital of Vienna, Vienna, Austria; ^11^Division of Rheumatology and Immunology, Department of Internal Medicine, Medical University Vienna, Vienna, Austria; ^12^Section for Outcomes Research, Centre for Medical Statistics, Informatics, and Intelligent Systems, Medical University Vienna, Vienna, Austria; ^13^Department of Rheumatology, Hospital of Brunico (SABES-ASDAA), Brunico, Italy

**Keywords:** Sjögren's syndrome, quality of life, PROMs, focus group technique, psychological impact, social impact, physical impact, ICF

## Abstract

**Objective:** To identify a spectrum of perspectives on functioning and health of patients with primary Sjögren's syndrome (pSS) from the five European countries in order to reveal commonalities and insights in their experiences.

**Methods:** A multicenter focus group study on the patients with pSS about their perspectives of functioning and health was performed. Focus groups were chaired by trained moderators based on an interview guide, audiotaped, and transcribed. After conducting a meaning condensation analysis of each focus group, we subsequently combined the extracted concepts from each country and mapped them to the International Classification of Functioning, Disability and Health (ICF).

**Results:** Fifty-one patients with pSS participated in 12 focus groups. We identified a total of 82 concepts meaningful to people with pSS. Of these, 55 (67%) were mentioned by the patients with pSS in at least four of five countries and 36 (44%) emerged in all the five countries. Most concepts were assigned to the ICF components *activities and participation* (*n* = 25, 30%), followed by 22 concepts (27%) that were considered to be *not definable* or *not covered* by the ICF; 15 concepts (18%) linked to *body structures and functions*. Participants reported several limitations in the daily life due to a mismatch between the capabilities of the person, the demands of the environment and the requirements of the activities.

**Conclusion:** Concepts that emerged in all the five non-English speaking countries may be used to guide the development and adaption of the patient-reported outcome measures and to enhance the provision of treatment options based on the aspects meaningful to patients with pSS in clinical routine.

## Introduction

Primary Sjögren's syndrome (pSS) is an autoimmune disease of unknown etiology with female predominance that is characterized by an inflammation of exocrine glands, particularly salivary and lachrymal glands as well as variable extra-glandular manifestations such as musculoskeletal, gastrointestinal, and/or neurological symptoms (1–3). Patients with pSS can experience multiple facets of the mucosal dryness, pain, fatigue, and other complaints resulting in an impairment in everyday life and an altered health-related quality of life (HRQoL) (4–9).

Perceptions of the disease may not only differ between the patients and health professionals (10, 11), but also between individuals suffering from the same condition (12). This is majorly due to that the perception is built upon several mental sources that are implicit and specific to the individuals (13). A recent study in the psoriatic arthritis (PsA) highlighted the influence of cultural backgrounds on differences in the perception of illness (14). The illness perception is dependent on the disease activity, and negative views of the illness are linked to the worse future health outcomes (15–17) whereas positive beliefs are associated with the better health outcomes (18–21).

Patient-reported outcomes (PROs) represent the “voice of the patient” and are increasingly applied in the research studies and clinical practice to ensure a patient-centered care (22, 23). PRO Measurements (PROMs) are the instruments to measure PROs. Several PROMs are available for pSS, however, only a few of them have been validated (24). Besides, the patients from non-English countries rarely participate in the studies aimed at the development or validation of PROMs leading to the underrepresentation of the specific cultural context of these countries in the final PROM (25). PROMs are therefore often cross-culturally adapted only later after their development (25, 26). However, the cross-cultural adaption of PROMs is linked with challenges such as not being familiar with different cultures or not considering geographical variations and differences in the physical and social infrastructure. Thus, cultural equivalence is not always achieved (25, 27–29).

An approach to truly capture what is important to the patients is qualitative methodology. It offers the opportunity to explore the perspectives, motivations, values, beliefs, and needs of the patients in a scientific and systematic manner. A qualitative approach supports the development of a deeper understanding of the patient perspective and enables the examination of complex issues that cannot be measured using strictly defined variables (30). The aggregation of views of the patients in a non-numeric, descriptive way can facilitate more patient-centered decisions (31) ultimately improving the quality of care.

Due to the increased relevance of the patient perspective (3), there is a growing body of qualitative evidence on the experiences of patients with pSS (7, 32–36). Thereby, the general impact of pSS on (health related) quality of life (33) or daily life (32, 36) was studied and specific aspects of the disease like physical, mental or ocular fatigue (34), fatigue, sleep, and pain (35), sleep disruption (7) or treatment experiences (32) were evaluated. However, all of these previous studies were conducted on a national level. None of them examined the perspectives of patients with pSS from different countries in one study so far, focusing on a consistent data collection and data analysis and taking potential cultural, geographical, and social variations into account.

In this study, the main objective was to identify a spectrum of perspectives on the functioning and health of patients with pSS in the five European countries in order to reveal commonalities and insights in their experiences.

## Patients and Methods

### Study Design

We conducted a qualitative multicenter study using the focus groups (37) in seven rheumatology centers in five European countries, namely, Austria, Germany, Italy, Romania, and Sweden. The “COnsolidated criteria for REporting QUalitative research” (COREQ)—guideline provided by the EQUATOR Network was used for the reporting of this study (38).

### Patients and Sampling

Patients with pSS had to meet the American–European Consensus Group Classification criteria, (6) and were recruited *via* telephone or face-to-face by the local investigators (BR, FB, TW, MD, MM, RD, CA, and PP) of the participating centers. Patients who already participated in another study at the same time or who had severe mental problems were excluded. Patients had to be fluent in the local language. We aimed to follow purposeful sampling by selecting patients of different ages and gender. However, pSS is predominantly affecting women (1–3, 39). According to the other qualitative studies in rheumatology (30, 40) and to balance the data quality and data quantity, we recruited two to four focus groups per country in order to gain data of enough richness.

### Data Collection

All focus groups were chaired by the trained moderators at the local rheumatology centers: JU (female, PhD student, occupational therapist) for Austria, Germany, and Italy, RD and CA (males, PhD, medical doctors) for Romania and MM (female, Ph.D., physiotherapist) for Sweden. We developed a common interview guide, which was translated into local languages, back translated, pilot tested, and refined. Interview questions used in this study are depicted in the supplement files (Supplementary Table S1). If necessary, field notes were taken during the focus groups by the moderators. Each focus group was audiotaped and transcribed in the local language. Ethical approval was obtained in each center, and all the participants provided oral and written informed consent.

### Data Analysis

The data analysis was carried out independently by the local researchers (JU for Austria, Germany, and Italy; RD for Romania; MM and CB for Sweden), using the method of meaning condensation (41). This involved the following four steps: first, local researchers read through all the focus groups transcripts and potential field notes of his/her center in order to become familiar with the data material. Second, specific units of a text, a few words, or a few sentences with a common meaning were identified in the data and defined as “meaning units”. Third, subconcepts contained in the “meaning units” were identified. A “meaning unit” could obtain more than one subconcept. Fourth, these subconcepts were grouped together to more comprehensive “higher-level” concepts, which were formulated by the local researchers in English language. A professional qualitative data analysis and research software known as Atlas.ti was used for the management of the data material (42).

After the focus groups were analyzed in each country, a one-day meeting with all researchers who conducted and analyzed the focus groups (JU, MM, and RD) was held. An experienced researcher (TAS) moderated this meeting, at which concepts from the five countries were compared and grouped together according to their meaning. Thereby, concepts representing the perspectives of the patients with pSS on functioning and health were identified. In order to describe these concepts in a standardized way with a common language, we used the International Classification of Functioning, Disability and Health (ICF) (43). This integrative biopsychosocial model, created by the WHO, is a highly recognized framework for classification that enables the description of problems of the patient, the selection of important outcome domains or the comparison of health information (43, 44). Hereinafter, two investigators (JU and AL) independently linked the concepts identified in the focus groups to the most precise ICF category according to the published linking rules (45). In case of any disagreement, the consensus was sought by discussion among the investigators.

### Rigor and Accuracy of the Qualitative Data Collection and Analysis

In order to ensure rigor and accuracy of the study, we followed several approaches. A detailed draft of the study protocol was available for all the study members prior to the beginning of the study. We ensured that the local researchers had the required knowledge and conducted an extensive training and debriefing session before data collection. Furthermore, we established a detailed track record for the data collection process that determined important conditions for conducting the focus groups. After data retrieval, a pilot analysis was done by each local researcher (CB, MM, JU, and RD) before the full proper analysis was conducted. The whole process was supervised by a researcher with extensive experience in the field of qualitative research (TAS). Both investigators (JU and AL) who conducted the ICF-linking process were trained in linking concepts to the ICF.

## Results

### Participants and Focus Groups

Demographic information from 51 patients with pSS who participated in 12 focus groups is provided in Table 1.

**Table 1:** Demographic data of the participants and characteristics of the focus groups per country.

**Demographic data and focus group characteristics**	**Austria**	**Germany**	**Italy**	**Romania**	**Sweden**	**Total**
Number of participants	8	16	6	11	10	51
Women, *n* (%)	8 (100)	15 (94)	6 (100)	11 (100)	10 (100)	50 (98)
Age (years), mean (±SD)	59 (±10.3)	57 (±14.9)	52 (±18.7)	65 (±10.1)	61 (± 9.2)	59 (± 13)
Disease duration (years), mean	9	6	8	7	16	9
**Marital status**, ***n*** **(%)**
Married	2 (25)	8 (50)	3 (50)	3 (27.3)	7 (70)	23 (45.1)
Cohabitant	4 (50)	1 (6.3)	2 (33.3)	0 (0)	1 (10)	8 (15.7)
Single	0 (0)	4 (25)	1 (16.7)	0 (0)	2 (20)	7 (13.7)
Divorced	1 (12.5)	1 (6.3)	0 (0)	3 (27.3)	0	5 (9.8)
Widowed	1 (12.5)	2 (12.5)	0 (0)	5 (45.5)	0	8 (15.7)
**Occupation**, ***n*** **(%)**
Employed	4	11	3	1	4	23 (45.1)
Self-Employed	0	1	0	0	0	1 (2)
Student	0	1	1	0	0	2 (3.9)
Unemployed	1	1	0	0	0	2 (3.9)
Retired	3	2	2	10	6	23 (45.1)
Number of focus groups	2	4	2	2	2	12
Number of participants per focus group, mean (range)	4 (3–5)	4 (3–6)	3 (3–3)	5.5 (5–6)	5 (5–5)	4.3 (3–6)
Interview duration (min), mean	112	94	73	63	81	86

*Because of the small sample size in the qualitative research and the focus on qualitative analysis in this study, no comparative statistics were calculated. We aimed to follow purposeful sampling by selecting patients with different age and gender. However, pSS is predominantly affecting women (1–3, 39)*.

### Concepts Identified in the Focus Groups and Their ICF Linkage

The local investigators (JU, MM/CB, and RD) identified 184 subconcepts in Austria, 156 in Germany, 151 in Italy, 98 in Romania, and 103 in Sweden. After the one-day-meeting of the local investigators, the analysis of all focus groups resulted in 82 concepts meaningful to the patients with pSS. Out of these 82 concepts, the largest number of concepts (*n* = 25, 30%) was linked to the ICF component *activities and participation*. The second largest number of concepts (*n* = 22, 27%) was considered to be *not definable* or *not covered* by the ICF. These concepts contained general facets of pSS such as the difficulty obtaining a diagnosis, the uncertainty of the origin of the disease or that being ill is patience and energy demanding. Fifteen (18%) concepts were linked to the ICF component *body functions and structures*, another 13 (16%) concepts expressed *environmental factors*. The remaining seven (9%) concepts revealed insights in the personal thoughts, coping styles and behavior patterns of patients with pSS and were therefore considered as *personal factors*. The ICF components of the concepts are shown in Tables 2–6 (column 2).

**Table 2:** Activities and participation.

**Concepts named by the people with pSS**	**ICF codes**	**A**	**G**	**I**	**R**	**S**
**Difficulty taking care of the own body and hygiene**, such as washing and brushing the hair, using deodorant, applying the body lotion all over the body	d510 Washing oneself d520 Caring for body parts	X			X	X
**Difficulty getting dressed** properly, including putting on pants, socks and shoes	d540 Dressing	X				X
**Reduced fine motor skills**	d440 Fine hand use	X			X	X
**Difficulty doing activities with hands and fingers**, such as opening a bottle, opening a jar, peeling vegetables, cutting with a knife, using the mobile phone or television remote control, needlework, manipulating jewelry, handling children's skiing boots or playing an instrument	d440 Fine hand use d445 Hand and arm use d9203 Crafts d550 Eating d630 Preparing meals d660 Assisting others d9202 Arts and culture d430 Lifting and carrying objects	X	X	X	X	X
**Difficulty managing the household**, including complex household work, preparing meals, cleaning the house or doing the shopping	d640 Doing housework d630 Preparing meals d650 Caring for household objects d6200 Shopping d6402 Cleaning living area		X	X	X	X
**Needing more time for the performance of activities, the difficulty of organizing job, daily life and therapy**	d2301 Managing daily routine d2303 Managing one's own activity level d299 General tasks and demands, other specified—needing more time for the performance of activities	X	X	X	X	X
**Difficulty performing (paid) work/education activities**, such as speaking, studying, writing, using the computer, working in front of a computer screen and following a discussion	d830 Higher education d850 Remunerative employment d8451 Maintaining a job d330 Speaking d170 Writing d3601 Using writing machines d355 Discussion	X	X	X	X	X
**Changed or lost employment due to disease, including not able to work full time**	d8509 Remunerative employment, unspecified–changed or lost employment due to disease d8501 Part-time employment	X	X	X	X	X
**Mobility problems and difficulty engaging in activities for leisure and recreation**, such as walking (the dog), hiking, driving a car, riding the motorbike, doing sports, dancing, traveling, gardening, going to the theater, watching TV or reading	d455 Moving around d450 Walking d6506 Taking care of animals, other specified—walking (the dog) d475 Driving d920 Recreation and leisure d9201 Sports d6505 Taking care of plants, indoors and outdoors d9202 Arts and culture d110 Watching d166 Reading	X	X	X	X	X
**Difficulty in keeping and changing the body position**, such as getting up in the morning, climbing the stairs, sitting on the floor for a long period of time, getting in and out of the car	d410 Changing basic body position d415 Maintaining a body position d4551 Climbing	X	X	X	X	X
**Decreased opportunity to join social activities**, such as going to a party, inviting friends at home, joining invitations for dinner	d9205 Socializing d9208 Recreation and leisure, other specified—going to a party d799 Interpersonal interactions and relationships, unspecified—joining invitations for dinner	X	X	X		X
**Caring for others is demanding**, including caring for children, partners, grandchildren or pets	d660 Assisting others d6506 Taking care of animals d660 Assisting others, unspecified—caring for others is demanding	X			X	X
**Impaired sex life**	d7702 sexual relationships	X				X
**Occupational loss**, such as “stopped hunting,” “stopped playing in the theater,” “not lying in the sun” or “skipped political work”	nd d910 Community life d9204 Hobbies, unspecified—lying in the sun d9204 Hobbies, unspecified—stopped hunting	X		X		X
**Some activities can make symptoms worse**, such as reading, watching TV, carrying heavy bags, going to work or doing intensive sport	d Activities and Participation, other specified—some activities make symptoms worse d110 Watching d166 Reading d850 Remunerative employment d430 Lifting and carrying objects d9201 Sports, unspecified—intensive sports	X		X	X	X
**Negative impact on relationships**, such as end of partnership/relationship or arguments with family members and friends	d720 Complex interpersonal interactions d770 Intimate relationships d750 Informal social relationships d760 Family relationships	X	X	X	X	X
**Balanced diet** influences well-being, such as eating no gluten, sugar, tomatoes and drinking no milk	d5701 Managing diet and fitness e1100 Food, unspecified—no gluten, sugar, tomatoes and milk	X	X			X
**Regular meaningful exercises in order to feel well and strengthen the body**, such as yoga, singing, going for a walk	d Activities and Participation, other specified—regular meaningful exercises in order to feel well and strengthen the body d570 Looking after one's health d9201 Sports, unspecified—Yoga d332 Singing d450 Walking	X	X	X	X	X
**Leisure activities experienced as meaningful**, such as reading, traveling, listening to music, surfing in the internet, doing crafts	d Activities and Participation, other specified—leisure activities experiences as meaningful d920 Recreation and leisure, unspecified—leisure activities experiences as meaningful d9204 Hobbies d920 Recreation and leisure, unspecified—surfing in the internet d115 Listening d9230 Crafts	X	X	X	X	X
**Everyday and self-care activities experienced as meaningful**, such as baking, cooking, doing the household and drinking morning coffee	d5 Self-care, other specified—everyday and self-care activities experienced as meaningful d6 Domestic life, other specified—everyday and self-care activities experienced as meaningful d630 Preparing meals d630 Preparing meals, unspecified—baking d640 Doing housework d560 Drinking, unspecified—drinking morning coffee	X	X	X	X	X
**(Paid) work and lifelong learning experienced as meaningful**	d8508 Remunerative employment, other specified—(paid) work experiences as meaningful d838 Education other specified—lifelong learning experiences as meaningful	X	X	X	X	X
**Co-existence and activities with others are experienced as meaningful**, such as engaging in gatherings with others, spending time with partner and friends, being a member of an association or joining a society	d9 Community, social and civic life—other specified, Co-existence and activities with others are experienced as meaningful d9205 Socializing d9100 Informal associations d9101 Formal associations	X	X	X	X	X
**Importance of rest, sleep and making breaks to manage daily life**	d2303 Managing one's own activity level	X	X	X	X	X
**Self-management strategies to reduce symptoms**	d570 Looking after one's health	X	X	X		X
**Looking for information and getting knowledge** about the disease and drugs, including reading books, obtaining information	d138 Acquiring information	X	X	X		

*This table shows the commonalities and insights in the lifeworld providing concepts meaningful to people with pSS as well as the corresponding ICF codes. ICF, International Classification of functioning, disability and health; nd, not defineable*.

*Countries in which we identified the concept were marked with X. Countries: A, Austria; G, Germany; I, Italy; R, Romania; S, Sweden*.

**Table 3:** Not covered, not defineable.

**Concepts named by the people with pSS**	**ICF codes**	**A**	**G**	**I**	**R**	**S**
**Uncertainty about the origin of the disease, the symptoms and discomforts**	nd-dis	X	X	X	X	X
**Being ill is patience- and energy demanding**, including insecurity due to having a chronic disease	nd-dis	X	X	X		X
**Factors that intensify emotional problems**, such as being alone, extreme fatigue, unpredictability of the disease and other environmental factors	nd- mh	X	X	X		X
**Side effects of drugs or intolerance toward drugs**, including loss of hair, hearing problems, nausea, diarrhea, fatigue, feeling dizzy	nd b850 Functions of hair b240 Sensations associated with hearing and vestibular function b5350 Sensation of nausea b525 Defecation functions b4552 Fatiguability b2401 Dizziness	X	X	X	X	X
**Factors that increase the dosage of medication**, such as “changing weather,” “certain activities,” and “severity of disease”	nd	X				
**Financial burden of the disease**, including a missing financial support from insurance system	nc e570 Social security services, systems and policies e5802 Health policies	X	X	X	X	X
**Time (and economy) limit** for recreational activities	nc d230 Carrying out daily routine, other specified—time limit e1650 financial assets, other specified—economy limit		X	X	X	X
**Co-morbidities and susceptible to other disease** like pneumonia, fungal infection or skin problems	nc-hc	X	X	X	X	X
**Ups and downs of symptoms** (swelling, stiffness, fatigue and dryness), including changes over the day and the general unpredictability of the disease	nc	X	X	X	X	X
**Lack of user-friendly information with the risk for misjudging the available information**	nc	X	X	X		
**Insufficient time with health personnel at the doctoral visits**	nc	X	X	X	X	
**Frequent doctoral visits cause stress and remind on being sick**	nc		X	X		
**Long travel distances and waiting times for a doctoral appointment inhibit regular doctoral visits or joining support groups**	nc	X	X	X		
**Invisibility of disease is either a relief or a curse**	nc	X	X			X
**Explaining the disease is important, but challenging**	nc	X				X
**Influenced roles within relationship, partnership and family life**	nc		X			X
**Difficulty obtaining a diagnosis**, including a great variability of the way until diagnosis	nc	X	X	X	X	X
**Nothing to worry, symptoms are in relation to age**	nc	X	X	X		X
**Presenteeism** due to fear of losing the job or sense of duty	nc		X			
**Knowing other people with disease causes a comfortable feeling**	nc	X	X	X	X	X
**Adaption of activities to own abilities**, such as changing the way of performing the activities or using other items	nc	X	X	X		X
**Having knowledge about the own abilities, the disease and its consequences for life cause a comfortable feeling and is necessary for adaptions in daily life**	nc	X	X	X		X

*This table shows the commonalities and insights in the lifeworld, providing concepts meaningful to people with pSS as well as the corresponding ICF codes. ICF, International Classification of functioning, disability and health; nc, not covered, nc-hc, not covered—health condition; nd, not defineable; nd–dis, not defineable—disability in general; nd–mh, not defineable-mental health*.

*Countries in which we identified the concept were marked with X. Countries: A, Austria; G, Germany; I, Italy; R, Romania; S, Sweden*.

**Table 4:** Body functions and structures.

**Concepts named by the people with pSS**	**ICF codes**	**A**	**G**	**I**	**R**	**S**
**Disturbed sleep**, either due to disease-related symptoms or due to side effects of coping strategies against dryness (e.g. frequent toilet visits as consequence of drinking a lot)	b134 Sleep functions	X	X	X	X	X
**Dryness- or disease related impact on body parts**, such as red/inflamed/sensitive/watery eyes, mouth, lymph nodes, blocked nose, nose bleeding, sicca, affected joints, skin/dental/vaginal problems	s220 Structure of eyeball s230 Structures around eye b210 Seeing functions b215 Functions of structures adjoining the eye b220 Sensations associated with the eye and adjoining structures s320 Structure of mouth s510 Structure of salivary glands s3200 Teeth b5104 Salivation b2153 Functions of lachrymal glands s4201 Lymphatic nodes b4353 Functions of lymph nodes s310 Structure of nose b255 Smell function b898 functions of the skin and related structures b710 Mobility in joints b28016 Pain in joints s6303 Structure of vagina and external genitalia b6709 Sensations associated with genital and reproductive functions, unspecified—vaginal problems	X	X	X	X	X
**Dryness-or disease related impact on functions**, such as an impaired ability to cry, ability to swallow, ability to dose salivary flow, ability to speak and sing, hearing and seeing problems	b340 Alternative vocalization functions b2153 functions of lachrymal glands b5105 Swallowing b5104 Salivation b310 Voice functions d330 Speaking b230 Hearing functions b210 Seeing functions	X	X	X	X	X
**Experiences of dryness and pain**, such “having vinegar in the yes,” “crackling voice,” “pelvis is like a wooden frame” or “my body bursts”	b280 Sensation of pain b220 Sensations associated with the eye and adjoining structures b3108 Voice functions, other specified—crackling voice b7808 Sensations related to muscles and movement functions, unspecified—pelvis is like a wooden frame	X	X	X	X	X
**Pain and aches** in the whole body and/or specific body parts	b280 Sensation of pain	X	X	X	X	X
**Fatigue and feeling exhausted** and their quantity and quality, such as “extreme/enormous fatigue”	b4552 Fatiguability	X	X	X	X	X
**Headache and heavy body** due to fatigue	b28010 Pain in head and neck b780 Sensations related to muscles and movement functions b4552 Fatiguability		X		X	
**Lack of energy** and **endurance**	b130 Energy and drive functions b4550 General physical endurance	X	X	X	X	X
**Experiences with other sensations like cramps or paraesthesia**, such as “like 1,000 ants running over the body”	b780 Sensations related to muscles and movement functions b735 Muscle tone functions b2702 Sensitivity to pressure		X	X		X
**Swelling** and **stiffness** affecting the whole body or specific body parts	b780 Sensations related to muscles and movement functions b7800 Sensation of muscle stiffness b545 Water, mineral and electrolyte balance functions	X	X	X	X	X
**Decreased memory and attention functions**, such as forgetfulness, troubles with finding the words and poor concentration	b140 Attention functions b144 Memory functions	X	X	X	X	X
**Emotional problems** like sadness, depression, worries, anger, anxiety, embarrassment, guilty feelings guilty feelings, fear and/or having suicidality thoughts and self-injury behaviors	b152 Emotional functions b1263 Psychic stability	X	X	X	X	X
**Reduced self-esteem, feeling odd and bored**	b1800 Experience of self b1801 Body image	X	X			X
**Hands become white and blue** when it is cold (indoor and outdoor)	b415 Blood vessel functions			X		X
Thoughts about **getting pregnant or thoughts during pregnancy**	b6601 Functions related to pregnancy		X	X		

*This table shows the commonalities and insights in the lifeworld, providing concepts meaningful to people with pSS as well as the corresponding ICF codes. ICF, International Classification of functioning, disability and health. Countries in which we identified the concept were marked with X. Countries: A, Austria; G, Germany; I, Italy; R, Romania; S, Sweden*.

**Table 5:** Environmental factors.

**Concepts named by the people with pSS**	**ICF codes**	**A**	**G**	**I**	**R**	**S**
**Hypersensitivity to environmental factors**, including spicy and sour food, alcohol, sparking beverages, coffee, bad air, sun (light), and water in the eyes, rooms with low humidity or air conditioning, extreme weather, make up, stress, stressful surroundings and noise	nc e1100 Food, unspecified—spicy and sour food e1100 Food, unspecified—alcohol e1100 Food, unspecified—sparkling beverages e1100 Food, unspecified—coffee e260 Air quality e225 Climate b21020 Light sensitivity e240 Light e1150 General products and technology for personal use in daily living, unspecified—make up e250 Sound	X	X	X	X	X
**Efficacy of drugs**, including good, limited or no effect	e1101 drugs, other specified—efficacy of drugs	X	X	X		X
**Not taken seriously and not understood by health personnel**, including a lack of counseling and understanding about what is important to patients	e450 Individual attitudes of health professionals e355 Health professionals e455 Individual attitudes of other professionals	X	X	X	X	X
**No access to wlan or living on the countryside** is negative for developing knowledge about the disease	e5608 Media services, systems and policies, other specified—No access to wlan or living on the countryside is negative for developing knowledge about the disease	X				
**Attitudes of employer**, including the employers (missing) duty of care toward employees	e430 Individual attitudes of people in positions of authority	X	X			X
**Being forced to paid work because of the social insurance system or the physicians interpretation of it**	e5708 Social security services, systems and policies, other specified—Being forced to paid work because of the social insurance system					X
**Adaptions in or of the environment**, including an ergonomic workplace, having a high bed or wearing certain kind of shoes	e1351 Assistive products and technology for employment e1401 Assistive products and technology for culture, recreation and sport e1151 Assistive products and technology for personal use in daily living d175 Solving problems d163 Thinking	X				X
**(Missing) Support and understanding other people**, including family, partner, children, relatives, colleagues and lecturers	e310 immediate family e410 Individual attitudes of immediate family members e315 Extended family e415 Individual attitudes of extended family members e320 Friends e420 Individual attitudes of friends e325 Acquaintances, peers, colleagues, neighbors and community members e425 Individual attitudes of acquaintances, peers, colleagues, neighbors and community members	X	X	X	X	X
**Family, children, grandchildren and friends enrich life and increase well-being**	e399 Support and relationships, unspecified—Family, children, grandchildren and friends enrich life and increase well-being e310 immediate family	X	X	X	X	X
**Experiences with non-medical treatment, alternative medicine and products**, such as physical therapy, ayurveda, salt water bath, sauna, thermal springs, massage, pedicure and taking homeopathic drugs	e5, other specified—Experiences with non-medical treatment, alternative medicine and products, such as physical therapy, ayurveda, salt water bath, sauna, thermal springs, massage, pedicure and taking homeopathic drugs	X	X		X	X
**Animals increase well-being**	e2201 Animals e2208 Fauna and flora, other specified—Animals increase well-being		X	X	X	X
**Nature as an important factor for health, including being active or passive in the nature**	e2200 Plants e2208 Fauna and flora, other specified—Nature as an important factor for health	X	X	X		X
**Looking for assistive devices and products that help**	d5702 Maintaining one's health, unspecified—looking for assistive devices and products that help e1151 Assistive products and technology for personal use in daily living e1401 Assistive products and technology for culture, recreation and sport e1351 Assistive products and technology for employment	X	X	X		X

*This table shows the commonalities and insights in the lifeworld, providing concepts meaningful to people with pSS as well as the corresponding ICF codes. ICF, International Classification of functioning, disability and health; nc, not covered*.

*Countries in which we identified the concept were marked with X. Countries: A, Austria; G, Germany; I, Italy; R, Romania; S, Sweden*.

**Table 6:** Personal factors.

**Concepts named by the people with pSS**	**ICF codes**	**A**	**G**	**I**	**R**	**S**
**Own attitudes toward drugs and drug intake**, either “accepting them”, “feeling dependent”, “some drugs are more dangerous than others” or “having the desire to stop taking them”	pf	X	X	X		X
**Difficulty to accept the disease and deterioration**, including a negative description	pf d130 Copying, unspecified—difficulty to accept the disease and deterioration		X	X		X
**(No) Openess with which people deal with their disease and their limitations**, including “telling others about the disease” or “not telling others and ”concealing the disease”	pf	X	X	X		X
**Being selfish and taking time for myself**	pf	X	X	X	X	X
**Prioritizing and performing activities that are meaningful and despite inner resistance**	pf	X	X	X	X	X
**Learned to live with the disease and try to live a normal life**, including accepting the disease and being positive	pf d130 Copying, unspecified—learned to live with the disease and try to live a normal life	X	X	X	X	X
**Personal beliefs and spirituality**	pf d9301 Spirituality				X	X

*This table shows the cross-cultural similarities and differences of concepts meaningful to people with pSS as well as the corresponding ICF codes. ICF, International Classification of functioning, disability and health; pf, personal factor*.

*Countries in which we identified the concept were marked with X. Countries: A, Austria; G, Germany; I, Italy; R, Romania; S, Sweden*.

### Commonalities and Insights in the Lifeworld of People With pSS

Out of 82 concepts, 55 (67%) were mentioned by the patients with pSS in at least four of five countries and 36 concepts (44%) emerged in all the five countries. Tables 2–6 gives an overview of the 82 concepts meaningful to the patients with pSS with their specific ICF codes.

Participants from all the countries reported several limitations in daily life, including self-care, productivity, and leisure. The reasons for these limitations are characterized by a mismatch between the capabilities of the person, the demands of the physical, social, and cultural environment, and the requirements to conduct the activity. A participant in Austria explained the impact of her decreased fine motor skills on the performance of activities as follows: “…in the morning, opening milk bottles or juice, that was impossible (…), but for example I cannot do anything with buttons, I cannot make the buttons, jewelry. I cannot do the little things. (…) you have to look what you wear, I want to put on a nice blouse, but I cannot do this if he (husband) is not at home because of the buttons.” Another example is given by a participant in Italy who described her experience with her decreased ability to remember things: “I am no longer receptive, I was a great student in high school, but when I started at the university (…), I was not able to remember things. I do not know what was happening, you know, from 1 day to the other, I just did not have the ability to concentrate anymore. When I attended lectures for 4 h in the morning, I did not know what was going on.”

How the environment can lead to participation restrictions was mentioned in all countries. One participant in Sweden clearly described: “Everybody else can go to town to go shopping, I cannot go inside (in the store) because of those big fans at the entrance, it does not work, my eyes do not manage it (the flowing air) at the entrance. So I am left outside waiting in the car.”

The importance of social support and understanding from other people was highlighted by the participants from all the five countries. In addition, the participants mentioned comfortable feeling that is created by knowing other people with the same disease. A participant in Austria explained it in the following way: “I think you meet so many friends and gossip with them about something. And you can't deal with your own problems with others, because they have no idea. So you can meet people who are concerned, and if you just have a coffee and chat…”.

However, it was not always possible to join support groups due to long travel distances. Some patients with pSS described the negative impact of long travel distances that inhibit them from visiting a healthcare provider or joining the support groups. Similar, but different is the concept about the negative impact of having no access to the internet or living on the countryside for the development of knowledge about disease.

Patients with pSS explained that they not always felt taken seriously and understood by the health personnel. One participant in Austria described it as follows: “…when you go to the dentist, he doesn't care (…), your whole mouth hurts anyway, because you don't have any saliva, and then he gives you the cotton wool, which is not ideal if you don't have any saliva. If he takes it out, he'll rip half of your skin with it. And then you say to him already five times ‘Listen'. And if he is not aware of Sjögren and I say, 'Listen, I have a dry mouth, please, watch out!', but he doesn't care.” At the same time the participants from four countries mentioned that the time with health personnel at routine visits is not sufficient. Furthermore, they specified the absence of user-friendly information and pointed the risk of misjudging. However, the importance of having knowledge about the own abilities, the disease and its consequences for life was found to be meaningful by the patients with pSS of the four countries.

## Discussion

To our knowledge, this is the first qualitative study on the European level that examined the perspectives of patients with pSS. We gained insights into the experiences of the patients with pSS from the Northern (Sweden), Central (Austria and Germany), and Southern/Eastern Europe (Italy, Romania). The results of our study highlight the importance of multinational data collection and go beyond previous small samples sized national studies, showing that the patients with pSS from different countries seem to experience similar challenges regarding their functioning and health. Concepts that expressed *body functions and structures* as well as concepts that were linked to *activities and participation* may be universal across the different European countries and might be used to guide the development and the cross-cultural adaption of the PROMs in pSS.

Our study led to the identification of 82 concepts; however, the largest number of concepts (*n* = 25, 30%) expressed aspects of an everyday living. This is in contrast to a study that aimed at exploring the perspectives of the patients with pSS on a national level (33), in which the concepts most often reported expressed the physical dimension of the disease. These differences may be explained by the multidimensional impact of pSS and the dynamic interaction between pSS, the affected body structures and functions and also the limitations in activities and restrictions in participation in relation to the personal and environmental factors.

Among the concepts found, the majority (67%) were mentioned by the people with pSS in at least four of five countries, and nearly half of the concepts (44%) were common in all the five countries. A similar observation was made in a recent study in which people with PsA from two different countries had two thirds of categories in common (14). In our study, two thirds of concepts assigned to *body functions and structures* (67%) and nearly half of the concepts related to the *activities and participation* (52%) were mentioned in all the five countries, indicating the importance of those two domains.

Some findings of our study are in line compared with other qualitative studies in pSS. We already know that the patients with pSS are confronted with a long way until diagnosis and thoughts about the origin of the disease and also the consequences of the disease on the physical, emotional, and social level (7, 33, 35, 36, 46, 47). We also know from other studies in pSS (33) and other rheumatic conditions that the patients sometimes feel that they are not taken seriously or that their complaints are dismissed by the health professionals (33, 46). The participants of our study specifically felt that the time the health personnel spent with them was insufficient. This is a notable result and should be seen in the context of ongoing debates about the future workforce requirements in rheumatology (48, 49). Current length of visits per patient and the time spent on the clinical care seem to be too short from point of view of the patients. Given that we expect an increasing lag between workforce supply and demand in the rheumatology in future, new concepts of care of pSS and other rheumatic patients might be needed.

Furthermore, attitudes toward drugs varied substantially between the participants of this study, either accepting them or feeling independent and having the desire to stop taking them. We do know from other studies that the attitudes toward taking drugs influence the adherence of the patients (50). Therefore, the health professionals should pay more attention to this point in order to ensure patient-centered care for the patients with pSS, also addressing non-pharmacological aspects and self-management strategies.

Our study has several limitations. First, we included a few European countries only. Considering the influence of social, cultural, and physical contexts into account, results might have been somewhat different if people from the other continents had participated in the study. On the other hand, we observed only few variations between the five countries (belonging to the Northern, Central, and Southern/Eastern Europe) studied, supporting the generalizability of our data.

Second, some concepts could have been influenced by the age, comorbidities, and other factors. Stratification of the focus groups according to these variables could have helped to measure the influence of these factors on the importance of the individual concepts. On the other hand, such an approach bore the risk that concepts emerged that are relevant for a small subgroup of the patients only. Our goal was to collect data representing the opinion and concerns of patients with pSS of everyday clinical practice and decided therefore against selecting patients according to the prespecified characteristics.

Third, the individual sample size in each country except Germany could have been bigger, however, saturation within the countries was reached that strengthens our results. We want to emphasize that the transferability of the identified lived experiences and perspectives is still limited, but a first attempt to investigate pSS with a cross-cultural understanding in order to inform practice and policy.

A specific strength of our study is that the health professionals with different occupations (occupational therapists, physicians, and physiotherapists) were involved in setting up the interview questions, analyzing and interpreting the data.

In conclusion, concepts meaningful to the patients with pSS identified in the five European countries might be used to guide the development and the adaption of PROMs. Concepts identified in this study enhance the clinical routine of the health professionals in order to provide support and treatment options based on the aspects relevant to the patients with pSS. However, the results of our study have to be considered preliminary and need to be confirmed by the future research.

## Data Availability Statement

The datasets presented in this article are not available because participants of this study did not provide consent for the sharing of the transcripts of the focus groups. For further information please contact Julia Unger, julia.unger@fh-joanneum.at.

## Ethics Statement

The studies involving human participants were reviewed and approved by the Ethic Committees of each center: [EK 1653/2015, (Vienna, 8 September 2015), EudraCT/EOMpss/Sjögren Syndrom (Vienna, 24 February 2016), EA1/014/16 (Berlin, 28 April 2016), 2961-2015 (Hanover, 1 December 2015), 93-2015 (Italy, 16 September 2015), 27/16.12.2015 (Romania, 16 December 2015), and 2016/35-31Ö (Sweden, 15 March 2016)]. The patients/participants provided their written informed consent to participate in this study.

## Author Contributions

TS, CD, and JU were involved in the study conception. BR, CB, FB, JU, MD, MM, PP, RD, TS, and TW were involved in the recruitment of the patients. CA, JU, MM, and RD collected the data. AL, CA, CB, CD, JU, MM, RD, and TS contributed substantially to analysis and interpretation of the data. CB, CD, JU, MM, RD, and TS drafted the manuscript. All authors read and approved the final manuscript.

## Funding

This work was supported by a grant of the Austrian Association of Rheumatology and the Region Norrbotten. None of them were involved in the data collection, nor in the analysis or preparation of the manuscript.

## Conflict of Interest

The authors declare that the research was conducted in the absence of any commercial or financial relationships that could be construed as a potential conflict of interest.

## Publisher's Note

All claims expressed in this article are solely those of the authors and do not necessarily represent those of their affiliated organizations, or those of the publisher, the editors and the reviewers. Any product that may be evaluated in this article, or claim that may be made by its manufacturer, is not guaranteed or endorsed by the publisher.
