# Supplementary material for: The Experiences of Functioning and Health of Patients With Primary Sjögren's Syndrome: A Multicenter Qualitative European Study
Source: Front Med (Lausanne). 2021 Nov 18;8:770422. doi: 10.3389/fmed.2021.770422 (PMC8637170; doi:10.3389/fmed.2021.770422)
Supplement: Supplementary file 1 [file Table_1.DOCX]

Supplementary Material

**The Experiences of Functioning and Health of Patients with Primary Sjögren’s Syndrome: A Multicentre Qualitative European Study**

# Online Supplementary Table S1. Main questions of the interview guide

| **Interview questions** |
| --- |
| I would like to ask you to think about a typical, ordinary day. If you think about your everyday life, which activities do you perform during a day? |
| If you think of your everyday life, in which daily life activities do you experience difficulties in performance? |
| How does primary Sjögren’s Syndrome influence your cognitive skills? |
| How does primary Sjögren’s Syndrome influence your physical skills? |
| How does primary Sjögren’s Syndrome influence your emotional skills? |
| How does primary Sjögren’s Syndrome influence your mental skills? |
| How does the environment influence your life with primary Sjögren’s syndrome? |
| Which influence does primary Sjögren’s Syndrome have on your social environment? |
| Which aspects in your life are very meaningful for you? |
| Which thoughts cross your mind when you think of quality of life? |
